# Strong and Nonspecific Synergistic Antibacterial Efficiency of Antibiotics Combined with Silver Nanoparticles at Very Low Concentrations Showing No Cytotoxic Effect

**DOI:** 10.3390/molecules21010026

**Published:** 2015-12-28

**Authors:** Aleš Panáček, Monika Smékalová, Martina Kilianová, Robert Prucek, Kateřina Bogdanová, Renata Večeřová, Milan Kolář, Markéta Havrdová, Grażyna Anna Płaza, Joanna Chojniak, Radek Zbořil, Libor Kvítek

**Affiliations:** 1 Regional Centre of Advanced Technologies and Materials, Department of Physical Chemistry, Faculty of Science, Palacký University in Olomouc, 17 listopadu 12, 771 46 Olomouc, Czech Republic; monika.smekalova@centrum.cz (M.S.); KilianovaMartina@seznam.cz (M.K.); robert.prucek@upol.cz (R.P.); radek.zboril@upol.cz (R.Z.); libor.kvitek@upol.cz (L.K.); 2 Department of Microbiology, Faculty of Medicine and Dentistry, Palacký University in Olomouc, Hněvotínská 5, 775 15 Olomouc, Czech Republic; katerina.bogdanova@upol.cz (K.B.); renata.vecerova@fnol.cz (R.V.); milan.kolar@fnol.cz (M.K.); 3 Regional Centre of Advanced Technologies and Materials, Department of Experimental Physics and Physical Chemistry, faculty of Science, Palacký University in Olomouc, 17 listopadu 12, 771 46 Olomouc, Czech Republic; marketa.havrdova@upol.cz; 4 Department of Environmental Microbiology, Institute for Ecology of Industrial Areas, Kossutha 6, 40-844 Katowice, Poland; pla@ietu.katowice.pl (G.A.P.); chojniak.joanna@gmail.com (J.C.)

**Keywords:** silver nanoparticle, antibiotics, antibacterial, resistant bacteria, synergism, cytotoxicity

## Abstract

The resistance of bacteria towards traditional antibiotics currently constitutes one of the most important health care issues with serious negative impacts in practice. Overcoming this issue can be achieved by using antibacterial agents with multimode antibacterial action. Silver nano-particles (AgNPs) are one of the well-known antibacterial substances showing such multimode antibacterial action. Therefore, AgNPs are suitable candidates for use in combinations with traditional antibiotics in order to improve their antibacterial action. In this work, a systematic study quantifying the synergistic effects of antibiotics with different modes of action and different chemical structures in combination with AgNPs against *Escherichia coli*, *Pseudomonas aeruginosa* and *Staphylococcus aureus* was performed. Employing the microdilution method as more suitable and reliable than the disc diffusion method, strong synergistic effects were shown for all tested antibiotics combined with AgNPs at very low concentrations of both antibiotics and AgNPs. No trends were observed for synergistic effects of antibiotics with different modes of action and different chemical structures in combination with AgNPs, indicating non-specific synergistic effects. Moreover, a very low amount of silver is needed for effective antibacterial action of the antibiotics, which represents an important finding for potential medical applications due to the negligible cytotoxic effect of AgNPs towards human cells at these concentration levels.

## 1. Introduction

An ever-increasing resistance of bacteria to the effects of existing antimicrobial agents is currently one of the most important health care issues with serious negative impacts such as higher morbidity and mortality rates in patients with infections caused by multi-resistant bacteria [1,2]. Moreover, the development and spread of bacterial resistance through a mechanism based on transfer of genetic material from resistant bacterial cells via recombination processes (extra-chromosomal resistance) result in relentless spread of resistance to antibiotics independent of the consumption of antibiotics [3]. Given the fact that even rational antibiotic therapy cannot stop the growth and spread of bacterial resistance and can only slow and delay it, we are likely to witness the end of the antibiotic era in medicine.

An option to overcome bacterial resistance is the combination of selected penicillin antibiotics (e.g., ampicillin, amoxicillin or piperacillin) with bacterial β-lactamase inhibitors (clavulanic acid, sulbactam or tazobactam) [4,5,6]. However, such combinations of antibiotics and other substances blocking the defined bacterial mechanism of resistance are currently confronted with a marked increase in the resistance of numerous bacterial species, according to the European Antimicrobial Resistance Surveillance Network (EARS-Net) [7]. Thus, if a novel antimicrobial drug under development is to be effective and free from bacterial resistance it must act at several cellular levels and not specifically like “traditional antibiotics”. The effect of silver, either as a metal (nanoparticles) or in compounds, is known to be not specific at a single level but to influence many bacterial structures and metabolic processes at the same time. Silver nanoparticles (AgNPs) were shown to inactivate bacterial enzymes [8,9], disrupt bacterial metabolic processes [10,11,12] and the bacterial cell wall, accumulate in the cytoplasmic membrane, increase its permeability [9,13,14], collapse the plasma membrane potential [12], interact with DNA [8], and generate reactive oxygen species [15,16,17], which damage biomacromolecules [18]. Thanks to their multi-level mode of action, AgNPs destroy or inhibit the growth of pathogenic microorganisms, including highly resistant bacterial strains (from units to several tens of mg/L) [13,14,19,20,21,22]. Therefore, AgNPs can be considered as a suitable candidate for combinations with antibiotics, posing no risk of bacterial resistance. No relevant data describing bacterial resistance to AgNPs or inactivation of antibacterial action of AgNPs have been published. Bacterial resistance to silver is observed only with ionic forms of silver and was disclosed in the works of Silver [23,24]. Bacteria resistant to ionic silver originated from clinical environments [25] and also naturally occurring strains [26]. Besides reduction of Ag^+^ to a less toxic oxidation state, the probable Ag^+^ resistance mechanism involves an active efflux from the cell by either P-type ATPases or chemiosmotic Ag^+^/H^+^ antiporters [27,28,29]. However, bacterial resistance to AgNPs has not been proven yet.

The synergy between AgNP and various antibacterial agents has been already studied in several works. Potara *et al.*, investigated the antimicrobial activity of chitosan-coated nanoparticles [30]. This combination of antimicrobials showed synergistic effects against two strains of *S. aureus*. MICs of the composites were approximately ten times lower than those of AgNP and chitosan alone. Another capping agent, myramistine, increased the activity of AgNP against *E. coli* up to 20-fold [31]. A combined treatment of lactoferrin/xylitol hydrogel and silver-based wound dressings acts synergistically against biofilms of clinical wound isolates of MRSA and *P. aeruginosa* [32]. Synergy evaluated with the help of fractional inhibitory concentration (FIC) index was observed against different Gram-negative bacteria when AgNPs were mixed with the membrane-permeabilizing antimicrobial peptides polymyxin B and gramicidin S [33].

Recently, several studies have indicated that AgNPs may strengthen the antibacterial effects of antibiotics against both susceptible and resistant bacteria, either additively or synergistically. The additive effect was shown in antibiotics of different mode of action against various bacterial strains [34,35,36,37,38,39,40]. However, this is only true if the concentrations of antibiotics and AgNPs reach their own minimum inhibitory concentrations (MICs), that is concentrations at which antimicrobial activity is achieved by the tested antibiotics or AgNPs alone, without being combined. So far, the actual synergistic effect of antibiotics and AgNPs at concentrations below their own effectiveness (*i.e.*, below MICs) has only been shown in a few studies on antibiotics combined with AgNPs [36,41,42,43,44]. Brown *et al.*, showed a synergistic effect of not only AgNPs, but also gold nanoparticles functionalized with ampicillin, even against multi-resistant strains such as multiple-antibiotic-resistant isolates of *Pseudomonas aeruginosa*, *Enterobacter aerogenes* and methicillin-resistant *Staphylococcus aureus* [45]. Nevertheless these finding clearly suggest that it would be feasible to find an effective combination of an antibiotic and another antimicrobial with a multi-level mode of action, resulting in a synergistic antimicrobial effect allowing efficient inhibition of bacterial pathogens using significantly lower doses as compared with an antibiotic alone.

Consistent with that finding, this study is the first to provide a systematic analysis quantifying the synergistic effects of antibiotics classified into groups according to their mechanism of action in combination with AgNPs against numerous pathogenic microorganisms such as Gram-negative or Gram-positive bacteria, representing some of the most important pathogens currently encountered in medical practice. Moreover, the synergistic effect was confirmed for all antibiotics tested in this study, irrespective of their mode of action. Relative to higher concentrations of antibiotics equal to units of mg/L needed in the absence of AgNP in treatment of infections, the achieved MICs for such combinations of antibiotics (as low as 10^−4^ mg/L) and AgNPs (up to several mg/L) may decrease the toxic burden for patients if applied in clinical practice.

## 2. Results and Discussion

### 2.1. Synthesis of Silver Nanoparticles

**Figure 1:**
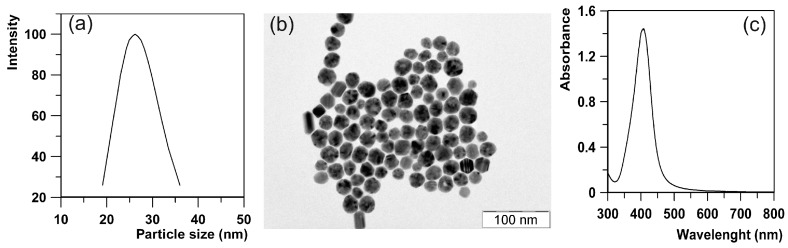
(**a**) Lognormal size distribution obtained from DLS measurements; (**b**) TEM image and (**c**) UV-Vis absorption spectra of AgNPs with diamater of 26 nm and narrow size distribution.

The modified Tollens process involving reduction of the [Ag(NH_3_)_2_]^+^ cation by d-maltose was used as a well-known, reliable and reproducible procedure for the preparation of AgNPs with a diameter of 26 nm and a narrow size distribution [46]. The size of 26 nm and narrow size distribution of the AgNPs documented by the lognormal size distribution curve obtained from DLS measurements were confirmed employing TEM (Figure 1a). In the UV-Vis absorption spectra of the synthesized AgNPs, a narrow surface plasmon absorption peak located at a wavelength of 410 nm also indicated the presence of nanometer-sized AgNPs (Figure 1c). Zeta potential value of −28 mV ensures high aggregation stability of such prepared AgNPs in aqueous dispersion. Non-aggregated and well separated AgNPs are seen in the TEM image (Figure 1b).

### 2.2. Synergistic Effect of Antibiotics and Silver Nanoparticles

The synergistic effect of AgNPs combined with antibiotics against both Gram-positive and Gram-negative bacteria, namely *Escherichia coli*, *Staphylococcus aureus* and *Pseudomonas aeruginosa*, was evaluated using the standard microdilution method to determine the MICs of the used antibiotics combined with AgNPs at different silver concentrations. For determination of the synergistic effects, AgNPs at very low concentrations of silver (under their MICs, *i.e.*, concentrations for which AgNPs did not exhibit antibacterial activity) were used against the tested bacteria to exclude any growth inhibitory effect of the AgNPs against the bacteria. The concentrations of silver used for the tests were 5, 2.5, 1.25 and 0.6 mg/L depending on the bacterial strain used. A broad spectrum of antibiotics was used in this study to observe whether the synergistic effect is specific or non-specific for antibiotics with different modes of action and different chemical structures. Therefore, in total more than 20 different antimicrobial substances of different modes of action and chemical structures were used (Table 1).

**Table 1:** The used antibiotics and their abbreviations divided into four groups based on their mode of action.

Inhibition of Cell Wall Synthesis	Inhibition of Protein Synthesis	Inhibition of Nucleic Acid Synthesis	Alteration in Cytoplasmic Membrane
Ampicillin (AMP) ^a^	Gentamicin (GEN) ^f^	Oxolinic acid (OXO) ^k^	Colistin (COL) ^m^
Ampicillin/sulbactam (AMS) ^a^	Tetracycline (TET) ^g^	Ofloxacin (OFL) ^k^	
Piperacillin (PIP) ^a^	Amikacin (AMI) ^f^	Ciprofloxacin (CIP) ^k^	
Piperacillin/tazobactam (PPT) ^a^	Chloramphenicol (CMP) ^h^	Co-trimoxazole (COT) ^l^	
Penicillin (PEN) ^a^	Erythromycin (ERY) ^i^		
Oxacillin (OXA) ^a^	Clindamycin (CLI) ^j^		
Cefazolin (CZL) ^b^			
Cefuroxime (CRX) ^b^			
Cefoxitin (CXT) ^b^			
Cefepime (CPM) ^b^			
Cefoperazone (CPR) ^b^			
Ceftazidime (CTZ) ^b^			
Meropenem (MER) ^c^			
Teicoplanin (TEI) ^d^			
Vancomycin (VAN) ^d^			
Aztreonam (AZT) ^e^			

Antibiotic categories based on their chemical structure: (^a^) penicillins; (^b^) cephalosporins; (^c^) carbapenems; (^d^) glycopeptides; (^e^) monobactams; (^f^) aminoglycosides; (^g^) tetracyclines; (^h^) amphenicols; (^i^) macrolides; (^j^) lincosamides; (^k^) quinolones; (^l^) sulfonamides; (^m^) polypeptides.

As seen from Table 2, Table 3 and Table 4 summarizing MICs of the used antibiotics alone and in combination with AgNPs, synergistic effects of antibiotics and AgNPs were shown for all antibiotics used and all bacterial strains. Strong synergistic effects of antibiotics and AgNPs against *Escherichia coli* were proven for silver concentrations of 5 and 2.5 mg/L. At these silver concentrations, the MICs of antibiotics were mostly two and three orders of magnitude lower than those of the non-combined (pure) antibiotics. For lower silver concentrations, the synergistic effect was observable for GEN, COL and OFL, with MICs more than twice as low as the MICs of the antibiotics alone. The fact that GEN, COL and OFL showed synergistic activity at the lowest silver concentration was apparently because of high susceptibility of *Escherichia coli* to these antibiotics. MICs of pure GEN, COL and OFL antibiotics against *Escherichia coli* were very low, under 1 mg/L. On the other hand, OXO and AZT also had MICs against *Escherichia coli* under 1 mg/L. However, the synergistic effects of these antibiotics were not observed at the lowest silver concentration. A very surprising and unexpected finding was observed in the case of the synergistic effect of AMP combined with AgNPs. AMP alone showed no antibacterial activity against *Escherichia coli*, even at the highest concentration equal to 32 mg/L, due to the resistance of *Escherichia coli* to this antibiotic. According to the European Committee on Antimicrobial Susceptibility Testing database, the AMP breakpoint against *Escherichia coli* is 8 mg/L and MICs higher than the breakpoint imply bacterial resistance [47]. It is evident from the obtained results that combination of AMP with AgNPs restores its antibacterial activity. A low concentration of silver is sufficient to restore the susceptibility of *Escherichia coli* to AMP.

The synergistic efficiency of antibiotics and AgNPs against *Pseudomonas aeruginosa* was similar to that against *Escherichia coli*. The MICs of antibiotics combined with AgNPs were two to three orders of magnitude lower for the highest silver concentrations (5 mg/L and 2.5 mg/L). In case of lower silver concentrations, the synergistic effect was observed for MER, GEN and COL (MIC more than twice as low when combined with AgNPs). The explanation is similar to that in the case of *Escherichia coli*; MER, GEN and COL are also antibiotics with high antibacterial activity showing low MICs against *Pseudomonas aeruginosa*.

**Table 2:** MICs of antibiotics (mg/L) in combination with AgNPs at different silver concentrations below the MIC of AgNPs (7.5 mg/L) against *Escherichia coli* CCM 4225.

	AMP	AMS	CZL	CRX	CXT	GEN	COT	COL	OXO	OFL	TET	AZT
ATB + 0 mg/L AgNPs	-	8	2	1	1	0.5	4	0.5	0.5	0.03	4	0.03
ATB + 0.6 mg/L AgNPs	-	8	2	1	1	0.03	2	0.125	0.5	0.015	4	0.03
ATB + 1.25 mg/L AgNPs	-	8	2	1	1	0.06	2	0.125	0.5	0.015	2	0.03
ATB + 2.5 mg/L AgNPs	0.03	0.03	0.0019	0.00097	0.00097	0.00048	2	0.00097	0.5	0.00024	1	0.0078
ATB + 5 mg/L AgNPs	0.00097	0.00097	0.00097	0.00097	0.00097	0.00048	0.0019	0.00048	0.00097	0.00024	0.00048	0.0019

**Table 3:** MICs of antibiotics (mg/L) in combination with AgNPs at different silver concentrations below the MIC of AgNPs (7.5 mg/L) against *Pseudomonas aeruginosa* CCM 3955.

	PIP	PPT	AZT	MER	CTZ	CPR	CPM	GEN	AMI	COL	OFL	CIP
ATB + 0 mg/L AgNPs	4	4	4	1	1	4	2	0.25	1	0.5	1	0.125
ATB + 0.6 mg/L AgNPs	4	4	4	1	1	4	1	0.03	0.25	0.125	1	0.03
ATB + 1.25 mg/L AgNPs	2	4	4	0.5	1	4	1	0.015	0.25	0.06	1	0.03
ATB + 2.5 mg/L AgNPs	0.015	0.0039	0.00097	0.0019	0.00024	0.00048	0.00024	0.00048	0.00048	0.00048	0.00024	0.00024
ATB + 5 mg/L AgNPs	0.0078	0.0039	0.00097	0.0019	0.00024	0.00048	0.00024	0.00048	0.00048	0.00048	0.00024	0.00024

**Table 4:** MICs of antibiotics (mg/L) in combination with AgNPs at different silver concentrations below the MIC of AgNPs (5 mg/L) against *Staphylococcus aureus* CCM 4223.

	PEN	OXA	AMS	CMP	TET	COT	ERY	CLI	CIP	GEN	TEI	VAN
ATB + 0 mg/L AgNPs	0.125	0.25	0.5	4	0.25	1	0.125	0.125	0.25	0.25	0.25	1
ATB + 0.6 mg/L AgNPs	0.015	0.25	0.125	4	0.015	0.03	0.06	0.015	0.03	0.25	0.03	0.03
ATB + 1.25 mg/L AgNPs	0.015	0.03	0.06	2	0.015	0.0078	0.06	0.015	0.015	0.06	0.015	0.00097
ATB + 2.5 mg/L AgNPs	0.00006	0.00048	0.00048	0.00048	0.00012	0.0019	0.00012	0.00012	0.00012	0.00048	0.00097	0.00048

*Staphylococcus aureus* was more susceptible to AgNPs than the other bacteria used in this work, with the MIC of AgNPs being 5 mg/L. The higher sensitivity of *Staphylococcus aureus* to AgNPs translated into stronger synergistic effects. The highest used silver concentrations (2.5 mg/L of silver) lead to the highest decrease of the MICs of the antibiotics, again of two to three orders of magnitude. With a silver concentration of 1.25 mg/L, synergistic effects, albeit not strong, were observed for nearly all tested antibiotics, with their MICs being decreased by approximately one order of magnitude. Indeed, unlike in *Escherichia coli* and *Pseudomonas aeruginosa* bacterial strains, the synergistic effect was more frequently observable at the lowest used silver concentration (0.6 mg/L). While the synergistic effects at the lowest used silver concentration were observable for only three antibiotics (GEN, COL and OFL and MER, GEN and COL) in case of *Escherichia coli* and *Pseudomonas aeruginosa*, there were synergistic effects for nine antibiotics in the case of *Staphylococcus aureus* (PEN, AMS, TET, COT, ERY, CLI, CIP, TEI and VAN), with their MICs being more than twice as low as MICs of the antibiotics alone. The reason for most tested antibiotics showing synergistic effects at the lowest silver concentrations is the low MICs of these antibiotics in the tested *Staphylococcus aureus* strain.

### 2.3. Cytotoxicity of Silver Antibiotics, Nanoparticles and Their Combinations

Cytotoxicity was evaluated for antibiotics selected from groups representing antibiotics with different modes of action. Overall ten antibiotics were tested separately and in combination with AgNPs to determine their LD_50_ toxicity index. Cytotoxicity of antibiotics and AgNPs was also determined at concentrations equal to their original MIC value obtained in the antibacterial assay (Table 5).

**Table 5:** Concentrations of silver NPs and antibiotics used in cytotoxicity determination.

AgNPs/ATB	Corresponding to MIC (mg/L)	Below MIC (mg/L)
Ag NPs	7.5	2.5
AMS	8	0.003
CZL	2	0.0019
MER	1	0.0019
CMP	4	0.00048
GEN	0.5	-
VAN	1	-
TET	4	-
CIP	0.25	-
COT	4	-
COL	0.5	-

**Figure 2:**
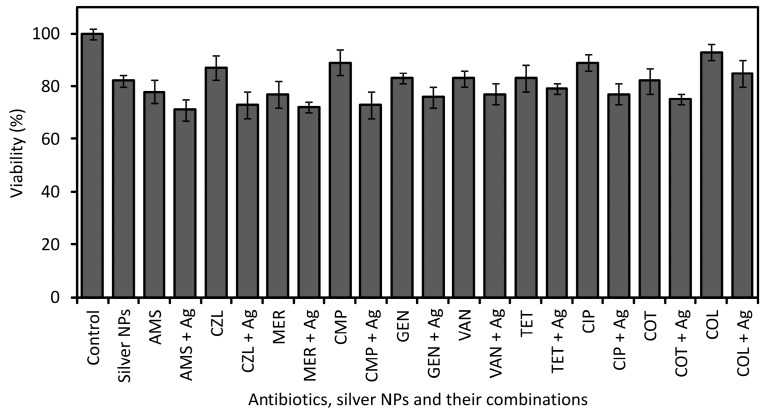
Viability of cells treated with antibiotics, silver NPs and their combinations using concentrations corresponding to the MIC value.

**Figure 3:**
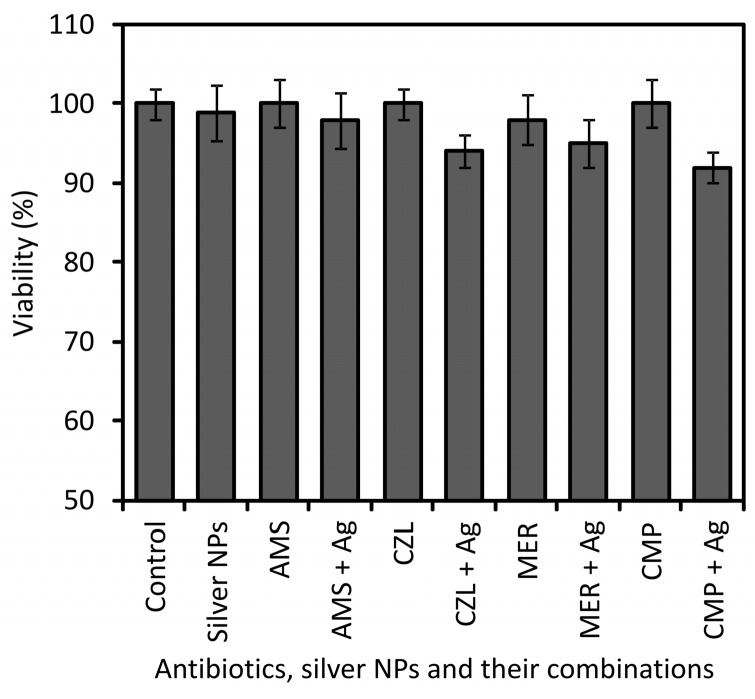
Viability of cells treated with antibiotics, silver NPs and their combinations using concentrations below the MIC value.

The LD_50_ values of the antibiotics themselves varied from 120 to 250 mg/L depending on the tested antibiotic (Table 6). When combined with AgNPs at concentrations of 7.5 mg/L and 15 mg/L respectively, the LD_50_ toxicity index of the antibiotics decreased and ranged from 90 mg/L to 180 mg/L and 80 mg/L to 150 mg/L, respectively. In the case of cytotoxicity evaluation at concentrations equal to MIC values, the antibiotics themselves only slightly inhibited the viability of cells; in most cases, the viability of cells treated with antibiotics decreased slightly to 90%–80% compared to that shown by untreated control cells (Figure 2). In the case of AMS and MER, the viability of cells was 78% and 77%, which still represents only a slight decrease in viability. Moreover, AgNPs themselves at the concentration corresponding to MIC only slightly inhibited the viability of cells down to 82% in comparison with control cells. The LD_50_ toxicity index of AgNPs was determined at the concentration of 30 mg/L. When antibiotics were combined with AgNPs, the viability of cells decreased from 85% to 71% compared to the control cells. The cytotoxic effect was higher as a result of the additive cytotoxic effects of the antibiotics and AgNPs. The highest cytotoxic effect was detected for the antibiotics AMS, CZL, MER and CMP combined with AgNPs; for those, the viability of cells decreased down to 71%, 73%, 72% and 73%, respectively. Combinations of antibiotics and AgNPs showing the highest inhibition of cell’s viability at concentrations equal to their MIC were used in further cytotoxicity tests at lower concentrations below their MIC, but still showing high antibacterial activity. Using these low concentrations of AMS, MER, CZL and CMP antibiotics and AgNPs themselves (not in combination), no inhibition of cell viability was observed in comparison with the control cells. When antibiotics were combined with silver NPs, the viability of cells only decreased to 90%–95% in comparison with the control cells depending on the antibiotics used (Figure 3).

**Table 6:** LD_50_ toxicity index (mg/L) of antibiotics alone and in combination with AgNPs at concentration of 7.5 mg/L and 15 mg/L respectively.

	AMS	CZL	MER	CMP	GEN	VAN	TET	CIP	COT	COL
ATB + 0 mg/L AgNPs	160	200	250	200	250	200	200	120	120	120
ATB + 7.5 mg/L AgNPs	100	130	180	150	180	150	180	100	100	100
ATB + 15 mg/L AgNPs	80	90	130	100	150	100	150	90	90	80

### 2.4. Discussion

**Figure 4:**
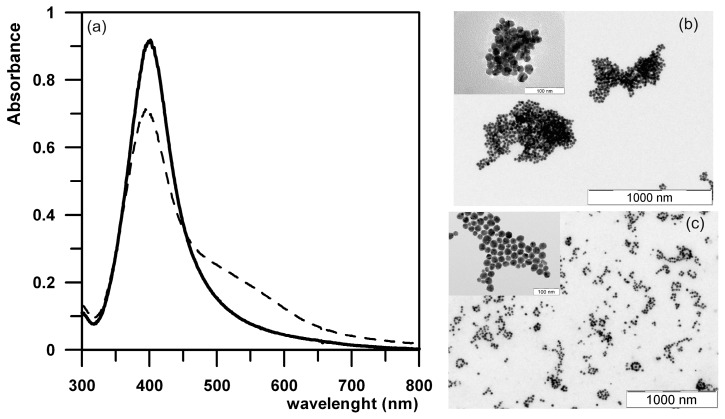
(**a**) UV-Vis absorption spectra of gelatin-stabilized (full line) and non-stabilized (dashed line) AgNPs dispersed in a culture medium at a ratio of 1:1; (**b**) TEM images of non-stabilized and (**c**) gelatin stabilized AgNPs.

AgNPs prepared using the modified Tollen’s process show a high aggregation and sedimentation stability themselves in aqueous dispersion (for up to several years) because of their high zeta potential value of −28 mV, ensuring sufficient repulsion between AgNPs. On the other hand, the prepared AgNPs had to be stabilized prior to the determination of synergistic effects with antibiotics because when non-stabilized AgNPs were added to the used culture media at a ratio of 1:1 (final silver concentration of 54 mg/L), partial aggregation was observed and monitored by DLS measurements and UV-Vis spectra. Aggregation of AgNPs and presence of aggregates lead to an increase in the average particle size from 28 to 85 nm. Also changes in absorption spectra typical for partial aggregation process of AgNPs such as a decrease in the absorption peak located at a wavelength of 410 nm (primary maximum) and emergence of a secondary absorption peak at 550 nm [19] were observed (Figure 4a). As well, the original negative zeta potential value of −28 mV decreased to −13 mV when AgNPs were mixed with the culture media. The decreases in the zeta potential value and subsequent partial aggregation process of AgNPs were induced by cations present in the culture media. Generally, AgNPs aggregate easily in environments with a high ionic strength, particularly when multivalent cations are presented. Oppositely charged cations are attracted to the AgNPs surface having negative surface charge and consequently decrease or eliminate completely their negative zeta potential. AgNPs with decreased or eliminated surface charge are not able to sufficiently repulse each other which lead to their approach, attachment, and consequently aggregation and sedimentation in media with a high ionic strength. The absorbance decrease of the primary maximum is viewed as a consequence of particle approach followed by their close joining and partial aggregation (Figure 4b). At the same time, a new secondary maximum peak emerged at a wavelength of 550 nm as a consequence of partial particle aggregation.

Therefore, AgNPs were stabilized with gelatin added at a very low concentration of 0.05% as an effective and non-toxic (natural) stabilizing substance. The gelatin was added after the synthesis of AgNPs (after the reduction process) so that gelatin did not affect this process and the particle characteristics of AgNPs such as size and size distribution. Such la ow gelatin concentration added after the synthesis of AgNPs has no effect on particle size as well as particle size distribution as confirmed by DLS and TEM measurements. The diameter of gelatin-stabilized AgNPs and their particle size distribution was unchanged compared to as-prepared non-stabilized AgNPs. No aggregation process occurred based on the results from DLS measurements, TEM characterization, and UV-Vis absorption spectrometry measurements when gelatin-stabilized AgNPs were mixed with culture media. The particle diameter of 26 nm and the location of the surface plasmon peak at a wavelength of 410 nm were unchanged when gelatin-stabilized AgNPs were added to the used culture media with no evidence of particle aggregation in the TEM images. TEM images of aggregated (non-stabilized) as well as non-aggregated AgNPs stabilized by 0.05% gelatin are shown in Figure 4. Non-stabilized AgNPs formed larger aggregates of several hundreds of nanometers in diameter as documented by the TEM images (Figure 4b). On the other hand, gelatin-stabilized AgNPs did not form any larger aggregates and they were still separated (Figure 4c). A control sample containing only 0.05% gelatin did not reveal antibacterial activity and the addition of the same amount of gelatin had no effect on the antibacterial efficiency of the antibiotics used.

No data have been previously published concerning such a strong synergistic effect of a broad spectrum of antibiotics and well characterized AgNPs at such low concentrations below their own MICs explored by the microdilution method. Most studies on the synergistic effects of antibiotics and AgNPs have been done using silver and/or antibiotic concentrations equal to or higher than their individual MICs. Moreover, this combined antibacterial effect was more additive than synergistic (multiple) and was not proved for all antibiotics involved in the studies [35,37,38,39,40]. The inhibition zone areas of the tested bacteria around discs containing particular antibiotics and AgNPs were in most cases only few millimeters larger. Moreover, the used disk diffusion method based on diffusion of antibiotic-AgNPs combinations in Muller-Hinton agar is not suitable and optimal given the lower diffusion ability of AgNPs which can result in a limitation of the synergistic effect. Only Gosh *et al.*, used AgNPs in concentrations lower than their own MICs in some cases combined with antibiotics at concentrations higher than their MIC and observed enhanced antibacterial activity for several combinations using the disc diffusion method [36].

In this work, the microdilution method was used for evaluation of synergistic effects of antibiotics and AgNPs as it is more suitable and reliable, especially with respect to specific properties of colloids and nanoscale materials such as diffusion and aggregation stability in dispersion media. The microdilution method is not limited by the diffusion ability of the tested compounds to the extent that the disk diffusion method is. Moreover, gelatin-stabilized AgNPs were stable and did not aggregate. Therefore, using this microdilution method, strong synergistic effects were shown for all tested antibiotics combined with AgNPs at very low concentrations of both antibiotics and AgNPs. On contrary, in the disk diffusion test, the aggregation stability of AgNPs can be affected after disk impregnation and also certain amounts of AgNPs may stay adsorbed on the paper disk. This can result in a lower antibacterial effect of the AgNPs and, given the limited diffusion of AgNPs, the final synergistic effect of antibiotics and AgNPs could be lower or non-existent.

Works using the microdilution method to prove the synergistic effects of antibiotics combined with AgNPs at concentrations under their own MICs were published by Li, Markowska and Singh [42,43,44]. Li reported preliminary results concerning the synergistic effect of amoxicillin and AgNPs against *Escherichia coli*. Although amoxicillin was not included in this study, the synergistic effect can be compared to that of ampicillin which is closely related to amoxicillin based on their similar chemical structure, mode of action and use for treatment of infections. Li *et al.*, reported high synergistic effects of amoxicillin combined with AgNPs at concentrations of 0.15 g/L of amoxicillin and 5 mg/L of AgNPs. In this study we report stronger synergistic effects of the related antibiotic ampicillin combined with AgNPs at lower concentrations of 0.03 mg/L of ampicillin and 2.5 mg/L of silver [42]. In the work by Singh, the enhancement of antibiotics’ efficiency combined with AgNPs was also proved only for β-lactam antibiotics [44]. In the work by Markowska, the synergetic effect was proved for ampicillin, streptomycin, rifampicin, and tetracycline [43]. Combinations of AgNPs with oxacillin, ciprofloxacin, meropenem, and ceftazidime showed no synergetic effect. In our study we proved a synergistic effect of a broader spectrum of antibiotics with different modes of action and chemical structures against bacterial strains used for microdilution tests. Moreover, our results clearly demonstrate that antibiotic-resistant bacteria become susceptible again when an antibiotic is combined with AgNPs as proved in case of the synergistic effect of ampicillin with AgNPs against *Escherichia coli*. This phenomenon was also recently published in articles by Singh and Brown [44,45]. Brown *et al.*, reported that AgNPs functionalized with ampicillin were effective against ampicillin-resistant *Escherichia coli* [45].

The mechanisms leading to enhancement in bacteria sensitivity towards antibiotics combined with AgNPs or even to restoration of sensitivity of bacteria originally resistant to antibiotics can be of various nature taking into account the multiple mode of action of AgNPs (AgNPs destroy bacteria by several mechanisms). AgNPs and antibiotics inhibiting synthesis of a cell wall can cooperate together promoting disturbance of the cell wall or direct damage of the cell wall. AgNPs could facilitate transport of hydrophilic antibitotics to the cell surface; increase in permeability of the membrane by AgNPs would help antibiotics to enter into the cells more easily. Inhibition of the activity of bacterial enzymes responsible for bacterial resistance could be another possible mechanism responsible for restoring of antibacterial activity of antibiotics. Enzymes produced by resistant bacteria such as β-lactamase, karbapenemase and others can be coated or bind on nanoparticle surfaces resulting in a change/modification of their structure. After that, enzymes become inactivated and enzymatic mediated hydrolysis of antibiotics cannot proceed. Enzymatic activity can be also inhibited by ionic silver released from AgNPs.

Another interesting and important fact observed in this work was that the synergistic effects of all used antibiotics in combination with AgNPs against both Gram-negative and Gram-positive bacteria were similar. Significantly higher or lower antibacterial effects against bacteria were observed for none of the used antibiotics and for none of the tested bacterial strains. Therefore, the difference in cell wall composition between Gram-negative and Gram-positive bacteria has no influence on the synergistic efficiency. No trends were observed for the synergistic effects of antibiotics with different modes of action and different chemical structures in combination with AgNPs against the tested bacteria, indicating non-specific synergistic effects of antibiotics in combination with AgNPs. It may be concluded that AgNPs do not affect bacteria by one specific mode of action such as damaging the bacterial cell wall or inhibiting proteosynthesis and nucleic acid synthesis which should result in stronger synergistic effects for antibiotics with certain modes of action. It is known that AgNPs change membrane permeability [48], collapse the plasma membrane potential [12], accumulate in the membrane and leads to formation of “pits“ in the cell wall [14]. Furthermore, AgNPs influence the metabolic processes of purine and inhibit enzymes such as tryptophanase or respiratory chain dehydrogenase thereby destroying important metabolic pathways of the bacteria [8,9,10,48]. Under aerobic conditions, silver ions are released from nanoparticles, they enter into the cells and subsequently generate reactive oxygen species (ROS), which damage DNA, RNA, proteins and lipids [18]. Zhang *et al.*, exploited this property and they developed nanostructured Ag/Cu composite with superior antibacterial activity. Metal oxides created in the thermal oxidation process on surface release silver ions more readily than zerovalent silver because of higher solubility of the oxides in water [49]. Due to their multiple modes of action, AgNPs attack bacteria by several mechanisms which lead to overall attenuation of the bacteria which cannot be so resistant to antibiotics. The non-specific antibacterial activity of AgNPs is important and significant for preventing the development of bacterial resistance to both AgNPs and preparations combining antibiotics and AgNPs.

It is known that the antibacterial activity of AgNPs strongly depends on their size, shape and surface modification by various capping agents [13,19,21,49,50,51,52,53]. The antibacterial activity of AgNPs increases with decreasing nanoparticle diameter [13,21,49]; anisotropic AgNPs such as triangular or hexagonal AgNPs show better antibacterial effect than spherical and rod-shaped AgNPs [51,52,53]. Surface modification of AgNPs by various capping agents such as polymer substances or surface active agents is generally used to enhance their aggregation stability or biological interactions with bacteria which consequently leads to enhancement of antibacterial activity of capped AgNPs. [19]. If these three key parameters (size, shape, and surface modification) directly influence the antibacterial activity of AgNPs themselves, they also similarly influence the synergistic effects of AgNPs combined with antibiotics. The role of size in synergistic effect of AgNPs combined with antibiotics was described in the works by Habash *et al.*, and Kareem *et al.* [54,55]. Habash *et al.*, evaluated synergistic effect using fractional inhibitory concentration index for 10 nm and 20 nm sized AgNPs combined with aztreonam against *Pseudomonas aeruginosa* where 40 nm, 60 nm, and 100 nm sized AgNPs showed no interaction when combined with antibiotic against the tested bacteria. [54]. Kareem *et al.*, also proved a better synergic antibacterial effect for 10 nm sized AgNPs compared to 20 nm sized ones combined with various antibiotics against *Staphylococcus aureus.* [55]. The effect of capping agents such as citrate, sodium dodecyl sulfate (SDS), and polyvinylpyrrolidone (PVP) on the synergistic effect of AgNPs combined with antibiotics was investigated in the work by Kora *et al.* [56]. Their results proved a synergistic effect for all the stabilized AgNPs combined with streptomycin, ampicillin, and tetracycline against Gram-negative and Gram-positive bacteria. The combined effect of capped AgNPs and antibiotics was more prominent with PVP-capped AgNPs as compared to citrate- and SDS-capped ones. Experimental data related to synergistic effects of AgNPs with various shapes combined with antibiotics have not been reported yet in the scientific literature, however, it can be presumed, based on the different antibacterial efficiency of AgNPs with various shapes that synergistic effect will also vary with AgNPs of different shapes.

The absence of adverse cytotoxic effects is important for potential medical applications of preparations or medical devices comprising combinations of antibiotics and AgNPs which could be used in treatment of local infections or production of antibacterial catheters, prostheses, vascular grafts *etc.* It may be assumed that prevention or treatment of infections would be more effective as strong synergistic antibacterial effects of antibiotics combined with AgNPs occurred at very low concentrations of both antimicrobial substances, minimizing the risk of side toxic effects. In this work, a very low concentration, equal to units or tenths of mg/L of both AgNPs and antibiotics themselves and in combinations, is needed for sufficient antibacterial effect; such low concentrations do not show cytotoxic effect to the NIH/3T3 cells. None of the tested antibiotics at concentrations corresponding to their MIC showed toxic effects. A toxicity index, LD_50_, was not proven for any of the tested antibiotics at a concentration equal to their MIC, and only a slight decrease of the viability in the range from 90% to 77% depending on the antibiotic used was observed. The LD_50_ toxicity index of antibiotics was determined at high concentrations ranging from 120 to 250 mg/L depending on the tested antibiotic. In addition, AgNPs themselves did not show cytotoxic effects to the tested cells at concentrations equal to their MIC (7.5 mg/L). The LD_50_ toxicity index of AgNPs was determined at the concentration of 30 mg/L. When antibiotics were combined with AgNPs and applied at concentrations equal to their MIC, the viability of the cells decreased from 85% to 75% compared to the control cells, except for the antibiotics AMS, MER, CZL, and CMP combined with AgNPs, for which the viability of the cells decreased down to 71%, 73%, 72% and 73%, respectively. In the case of AMS combined with AgNPs, the highest decrease in the viability to 71% do not reach the LD_50_ index and, thus, such a combination revealing the highest decrease in the viability does not show a cytotoxic effect. The LD_50_ toxicity indexes of antibiotics combined with 7.5 mg/L and 15 mg/L of AgNPs was determined at high concentrations ranging from 90 mg/L to 180 mg/L and 80 mg/L to 150 mg/L, respectively. When antibiotics were combined with AgNPs at concentrations below their MIC, which is still sufficient for strong antibacterial effect, the viability of the cells decreased very slightly to 90%–95% in comparison with the control cells, depending on the antibiotics used. Such a negligible inhibition of the cell’s viability is not regarded to have any cytotoxic effect.

AgNPs themselves at concentrations below 30 mg/L do not also display a cytotoxic effect to human cells or blood as well as to environmentally important organisms as was proved in earlier studies [20,57,58,59]. Moreover, Richter *et al.*, came up with a solution to the persistence of AgNPs in the environment and created nanoparticles with biodegradable lignin cores loaded with silver ions and coated with a cationic polyelectrolyte layer. Such nanoparticles lose their post-utilization antibacterial activity because of depletion of the silver ions, thereby minimizing their environmental impact [60]. In other study by Munger *et al.*, no *in vivo* toxicity effects of commercial AgNP to humans were observed. No clinically important changes in metabolic, urine, hematologic, physical findings or imaging morphology were noted after 14 days of exposure to 10 and 32 ppm of AgNPs, indicating that exposure to low doses of AgNPs has no adverse or toxic effects to humans [61]. Recently, several papers focusing on *in vivo* experiments evaluating toxicity and adverse effects of AgNPs using experimental animals have been published [62,63,64,65,66,67,68]. The obtained data related to the adverse and toxic effects of AgNPs vary strongly in each scientific paper and the final conclusions are still controversial. Generally, oral exposures to AgNP cause weight loss, inflammatory and immune responses, hepatic changes, increase neurotransmitter levels and changed blood values [62,63,66,67,68] in animal model experiments at concentrations of units or tens of mg/kg. Out of all the reports, the lowest observed adverse effect level expressed by increased cytokines concentration is 0.5 mg/kg of bw/day in mice following 28-day oral Ag NP exposure [68]. On the other hand, Kim *et al.*, observed only slight liver damage when rats were exposed to over more than 300 mg/kg of Ag [62]. Dermal toxicity studies indicate that exposure to >0.1 mg/kg of AgNPs results in slight spleen, liver, and skin damage in guinea pigs [69]. Tang *et al.*, examined the distribution and toxicity of AgNP in rats administered subcutaneous injections of silver at 62.8 mg/kg [70]. AgNPs were translocated to the blood circulation and distributed to kidney, liver, spleen, brain, and lung. Moreover, AgNP caused blood-brain barrier destruction and neuronal degeneration [71]. On the other hand, such an amount (62.8. mg/kg) of Ag in the case of subcutaneous injection or 300 mg/kg in the case of oral administration or even higher amounts represent extremely high doses which do not need to be administrated to treat bacterial infections, especially when AgNPs are combined with antibiotics. Unfortunately, the issue of the relevant dose of AgNPs required for system or local elimination of infection has not been addressed, verified or published yet. Similarly, pharmacological and pharmacokinetic data on AgNPs have not been described yet. Thus, it is not possible to predict in advance the doses of AgNPs and their eventual adverse effects. This is still an open field which requires further exploration in order to determine if AgNPs combined with antibiotics can be effective for the local and systematic therapy of infectious diseases without showing any side or adverse effects.

## 3. Experimental Section

### 3.1. Chemicals and Biological Materials

For the synthesis of AgNPs, silver nitrate (99.9%, Fagron, Olomouc, Czech Republic), ammonia (p.a., 25% *w*/*w*) aqueous solution, Sigma-Aldrich, St. Louis, MO, USA), sodium hydroxide (p.a., Lach-Ner, Neratovice, Czech Republic) and d-maltose monohydrate (p.a., Sigma-Aldrich) were used. The prepared AgNPs were stabilized by gelatin (p.a., Lach-Ner).

For the determination of MICs of tested antibiotics, AgNPs and their combinations, Mueller-Hinton broth (Becton, Dickinson and Company, Franklin Lakes, NJ, USA) was used. The following standard reference strains (labeling according to Czech Collection of Microorganisms, Masaryk University, Brno, Czech Republic) were used: *Escherichia coli* CCM 4225, *Pseudomonas aeruginosa* CCM 3955 and *Staphylococcus aureus* CCM 4223. Antibiotics involved in the study are summarized in Table 1.

### 3.2. Synthesis and Characterization of Silver Nanoparticles

For the purpose of this study, AgNPs with an average size of 28 nm and a very narrow size distribution were prepared by the well-established modified Tollens process involving reduction of the complex cation [Ag(NH_3_)_2_]^+^ by d-maltose in alkaline media (pH = 11.5) [46]. The initial concentrations of the reagents in the reaction system were as follows: silver nitrate 1 × 10^−3^ mol/L; ammonia 5 ×·10^−3^ mol/L; sodium hydroxide 1·10^−2^ mol/L and d-maltose as a reducing agent 1 × 10^−2^ mol/L. The reaction system was stirred continuously with a magnetic stirrer and the entire experiment was carried out at the laboratory temperature (approx. 21 °C). The prepared AgNPs were additionally stabilized by gelatin added to the prepared dispersion of AgNPs at a final concentration of 0.05% in order to prevent partial aggregation caused by the culture medium.

The diameter of the prepared AgNPs was measured by the dynamic light scattering (DLS) method with the 90 Plus Particle Size Analyzer (Brookhaven Instruments Co., Holtsville, NY, USA). The same instrument was used for zeta potential measurements. The nanodimensions of the synthesized AgNPs were confirmed using transmission electron microscopy (TEM) with the JEM 2010 (Jeol, Tokyo, Japan) instrument. UV-Vis spectra of ten-fold diluted dispersions of AgNPs were recorded with a Specord S 600 spectrophotometer (Analytik Jena, Jena, Germany).

### 3.3. Determination of the Synergistic Effect of Antibiotics and Silver Nanoparticles

MICs of antibiotics and AgNPs were separately determined by the standard microdilution method. The same approach was used to determine the synergistic effect of the tested antibiotics combined with AgNPs. The used antibiotics were diluted in geometric progression and combined with AgNPs at silver concentrations under the MICs of AgNPs against the tested bacterial strain. The used silver concentrations ranged from 0.6 to 5 mg/L according to the bacterial strain used. Disposable microtitration plates were used for the tests. A total of 100 μL of Mueller-Hinton broth with defined concentrations of an antibiotic and AgNPs was inoculated with the tested bacteria at a concentration of 10^5^ to 10^6^ CFU/mL. The MIC was read after 24 h of incubation at 37 °C as the MIC of the tested substance that inhibited the growth of the bacterial strain. Simultaneously, MICs of antibiotics and AgNPs were separately determined using the same microdilution method.

### 3.4. Cytotoxicity Evaluation of Antibiotics, Silver Nanoparticles and Their Combinations

Cytotoxicity was determined for AgNPs and selected antibiotics themselves and also for their combinations at concentrations showing antibacterial effect (equal to and under the MIC values). Also the toxicity index (LD_50_) of selected antibiotics and AgNPs themselves and for their combinations was evaluated. Procedure of cytotoxicity evaluation was slightly modified in order to exclude potential additive effect of antibiotics applied in standard procedure. Therefore the cells NIH/3T3 were cultivated in medium (DMEM, Life Technologies^TM^, Carlsbad, CA, USA) without supplemented antibiotics for three passages in order to obtain antibiotic-free cells. Subsequently, cells were seeded into 96-well plate (TPP, Biotech, Trasadingen, Switzerland) at a density of 2 × 10^4^ cells per well. After spreading (4 h), the cells were treated with the samples (antibiotics, AgNPs and their combination) and incubated in a fully-humidified incubator with 37 °C and 5% CO_2_ atmosphere. Following the treatment (24 h), the MTT solution (at concentration 5 mg/mL) was added (20 μL) into the each well. MTT solution was gently removed after 4 h and dimethyl sulfoxide (100 μL) was added to dissolve formazan crystals. Absorbance was read at 570 nm by multiplate reader Infinite PRO M200 (Tecan, Männedorf, Switzerland). Survival rate of cells was calculated from relation of absorbance: (A_sample_/A_control_) × 100 and plotted as percentage of viability. Each tested concentration was repeated three times and all experiments were carried out in duplicate.

## 4. Conclusions

In conclusion, we report a strong synergistic efficiency of a broad spectrum of antibiotics with different chemical structures and modes of action in combination with AgNPs against *Escherichia coli* CCM 4225, *Pseudomonas aeruginosa* CCM 3955 and *Staphylococcus aureus* CCM 4223. AgNPs of an average size of 28 nm with a narrow size distribution were synthesized by the modified Tollens process and stabilized by gelatin to achieve high antibacterial efficiency by preventing particle aggregation in culture media. The microdilution method of determining the MICs of antibiotics was used as a method that is more suitable and reliable than the disc diffusion method. The antibacterial activity of the tested antibiotics increased markedly when combined with AgNPs as was proved by the significantly decreased MICs of the antibiotics against the tested bacteria. The synergistic effect of antibiotics combined with AgNPs was proved at very low concentrations showing no cytotoxic effect on mammalian cells. Moreover, restoration of the susceptibility of a resistant *Escherichia coli* strain to ampicillin was observed when ampicillin was combined with AgNPs. Because of a lack of data concerning this phenomenon in the literature it is necessary to perform larger studies to investigate the possible restoration of susceptibility of resistant bacteria to antibiotics when combined with AgNPs.
